# Deciphering the genomic insights into the coexistence of congenital scoliosis and congenital anomalies of the kidney and urinary tract

**DOI:** 10.3389/fgene.2024.1399604

**Published:** 2024-07-23

**Authors:** Haojun Wang, Wen Wen, Mingxi Yao, Tongwang Yang, Dongshan Chen, Wei Wang

**Affiliations:** ^1^ Department of Urology, Beijing Chaoyang Hospital Affiliated Capital Medical University, Beijing, China; ^2^ State Key Laboratory of Complex Severe and Rare Diseases, Department of Orthopedic Surgery, Peking Union Medical College Hospital, Peking Union Medical College and Chinese Academy of Medical Sciences, Beijing, China; ^3^ Beijing Key Laboratory for Genetic Research of Skeletal Deformity, Beijing, China; ^4^ Key Laboratory of Big Data for Spinal Deformities, Chinese Academy of Medical Sciences, Beijing, China; ^5^ Chinese Academy of Medical Sciences and Peking Union Medical College, Beijing, China; ^6^ Academician Workstation, Changsha Medical University, Changsha, China; ^7^ Hunan Key Laboratory of the Research and Development of Novel Pharmaceutical Preparations, Changsha Medical University, Changsha, China; ^8^ Department of Urology, Qilu Hospital of Shandong University, Jinan, China

**Keywords:** congenital scoliosis (CS), CAKUT, mutational burden analysis, molecular etiology, *PTPN11*, CNV association

## Abstract

**Background:**

Congenital scoliosis and congenital anomalies of the kidney and urinary tract are distinct genetic disorders with differing clinical manifestations. Clinically, their coexistence is not rare, but the etiologies of these complex diseases remain largely unknown, especially their shared genetic basis.

**Methods:**

We sequenced the genomes of 40 individuals diagnosed with both CS and CAKUT, alongside 2,764 controls from a Chinese Han population cohort. Our analyses encompassed gene-based and pathway-based weighted rare variant association tests, complemented by copy number variant association analyses, aiming to unravel the shared genomic etiology underlying these congenital conditions.

**Results:**

Gene-based analysis identified PTPN11 as a pivotal gene influencing both skeletal and urinary system development (*P* = 1.95E-21), participating in metabolic pathways, especially the MAPK/ERK pathway known to regulate skeletal and urinary system development. Pathway-based enrichment showed a significant signal in the MAPK/ERK pathway (*P* = 3E-04), reinforcing the potential role of PTPN11 and MAPK/ERK pathway in both conditions. Additionally, CNV analysis pinpointed IGFLR1 haploinsufficiency as a potential influential factor in the combined CS-CAKUT phenotypic spectrum.

**Conclusion:**

This study enriches our understanding of the intricate genomic interplay underlying congenital scoliosis and kidney and urinary tract anomalies, emphasizing the shared genetic foundations between these two disorders.

## 1 Introduction

Congenital Anomalies of the Kidneys and Urinary Tract (CAKUT) encompass diverse kidney or urinary tract malformations, often leading to renal failure (Vivante et al., 2014; van der Ven et al., 2018). Most CAKUT cases are identified in early life through routine prenatal ultrasound examinations (Ramanathan et al., 2016). These malformations include kidney agenesis (KA), ectopic kidney or horseshoe kidney (EK-HK), obstructive Uropathy (OU), duplicated collecting system and/or ureter (DCS), other lower urinary tract malformation (LUTM), posterior urethral valves (PUV) and vesicoureteral reflux (VUR). CAKUT accounts for nearly half of all chronic kidney disease cases within the initial 30 years of life, resulting in substantial clinical challenges (Vivante et al., 2014).

Congenital Scoliosis (CS) is another congenital disorder characterized by lateral curvature of the spine (Feng et al., 2021), with the population incidence of less than 1%. Interestingly, the coexistence of the two congenital disorder was reported occasionally. A meta review suggested the incidence of genitourinary anomalies associated with congenital scoliosis was as high as 22.91% (Lorente et al., 2023). They can be part of a recognized syndrome or genetic condition, such as VACTRL syndrome (Lorente et al., 2023) and 16p11.2 microdeletion syndrome (Lorente et al., 2023). While in other cases, they may occur together by chance or due to unidentified genetic factors.

During human embryonic development, both primitive kidneys and the spine originate from intermediate mesoderm tissue. Activation of specific signaling pathways and genes prompt cell differentiation within the intermediate mesoderm, leading to primitive kidney structures and spine formation. These signaling pathways include Wnt, Notch and Bone Morphogenetic Protein (BMP) pathways, with BMP playing a crucial role in embryonic development, tissue regeneration, and skeletal morphology (McMahon et al., 1998; Kopp, 2000; Cain et al., 2005; Cheng and Kopan, 2005). Furthermore, the kidneys and spine share certain fundamental features, including the process of left-right rotation during development, ensuring their proper positioning and functionality within the body (Cartwright et al., 2004). These common characteristics highlight the intersecting developmental mechanisms integral to the formation of both the spine and the kidneys.

Our previous research demonstrated potential shared genetic mechanisms between CS and CAKUT (Li et al., 2022), indicating inadequate *TBX6* gene dosage could contribute to the coexistence. However, this can only explain a small part of the patients, and the comprehensive genetic basis of this coexistence remains largely unknown. In this study, we conducted next-generation sequencing on a Chinese Han population cohort affected by CS-CAKUT. Our aim was to analyze rare variants across the entire exome and their involvement in metabolic pathways, with the intention of unraveling the intricate genetic underpinnings of CS-CAKUT.

## 2 Materials and methods

### 2.1 Study subject

As of December 2022, among 2,764 patients undergoing treatment for congenital scoliosis at Peking Union Medical College Hospital, 40 Han Chinese patients were confirmed to have congenital kidney anomalies with associated urinary tract abnormalities. The phenotype evaluation for congenital scoliosis and congenital anomalies of the kidney and urinary tract was performed by different specialists in orthopedics and nephrology. Patients were categorized to different phenotypical subgroups. CS type I was characterized by simple vertebral dysplasia, CS type II by vertebral segmentation defects, and CS type III represented a combination of the previous two conditions. To note, severe infantile idiopathic scoliosis (IS) was also included. The diagnostic criteria have been reported in prior study (Li et al., 2022). Written informed consent was obtained from all participants at the time of recruitment. This study was approved by the Ethics Committee of Peking Union Medical College Hospital (I-22PJ976).

### 2.2 Next-generation sequencing (NGS)

Next-generation sequencing (NGS), encompassing both exome sequencing (ES) and genome sequencing (GS), was carried out on DNA samples extracted from peripheral blood of the study participants. ES was conducted in paired-end mode on the Illumina HiSeq 2000 platform, maintaining an average depth of coverage at 70X. To create paired-end libraries, three different capture kits were utilized: the xGEN targeted capture kit v2 (IDT, Coralville, IA, United States), VCRome SeqCap EZ Choice HGSC 96 Reactions (Roche, Pleasanton, CA, United States), and SureSelect Human All Exon V6 + UTR r2 core design (Agilent, Santa Clara, CA, United States).

For GS, sequencing libraries were generated following the manufacturer’s recommended protocol using the KAPA Hyper Prep kit (KAPA Biosystems, Kusatsu, Japan). Subsequently, PCR-free sequencing was executed on either the Illumina HiSeq X-Ten or NovaSeq platform, achieving an average depth of coverage of 35X.

### 2.3 Variant calling and filtering

The study employed the Peking Union Medical College Hospital pipeline for variant calling and quality control (Chen et al., 2021; Zhao et al., 2021). We utilized the XHMM tool to analyze copy number variations in whole-exome sequencing (WES) data and employed the CNVpytor tool to analyze copy number variations in whole-genome sequencing (WGS) data. Raw sequencing reads were aligned to the GRCh37 genome, and variants were identified using Sentieon software (Supplementary Methods). Following rigorous quality control of both cariant and sample level, 12,041 variants from 40 cases and 2,764 controls were retained (Supplementary Methods). In brief, this process involved hard filtering, population-based filtering, and Variant Quality Score Recalibration (VQSR). The final dataset underwent principal component analysis (PCA) and identity-by-descent (IBD) analysis for further refinement.

### 2.4 Variant annotation

The annotation of small variants (i.e., SNVs and indels) was carried out using the Variant Effect Predictor (VEP), which predicted the effects of the variants on transcript and protein sequences. The LofTee (https://github.com/konradjk/loftee) and dbNSFP plugins were used to generate bioinformatic predictions. Transcript-level information was annotated using the NCBI RefSeq database (https://www.ncbi.nlm.nih.gov/refseq/, accessed on 2022/05/25), with the RefSeq transcript labeled as “canonical” by ENSEMBL (https://www.ensembl.org/, accessed on 2022/05/25) being selected. CNVs were annotated using AnnotSV (v3.0), which compiled genomic and genetic information including genes and functional elements involved, gene-based annotations, genomic content around breakpoints, and overlapping records from population and disease databases.

### 2.5 Gene-based rare variant association analysis

The Genome Aggregation Database (gnomAD, https://gnomad.broadinstitute.org/) was utilized for annotating the population frequencies of variants. Rare variants with a gnomAD population-max allele frequency ≤ 0.1% and cohort allele frequency ≤ 0.1% were selected for gene-based genetic burden analysis. Subsequently, the retained variants were further annotated with transcript-level information from the NCBI RefSeq database (https://www.ncbi.nlm.nih.gov/refseq/). Each variant was categorized into different mask levels (from 1 to 6) and assigned weight values (ranging from 1 to 0) based on variant type and bioinformatic prediction results. The mutational burden of a given gene in each individual was designated as the maximum weight value among all ultra-rare variants carried by that individual. The ACAT package 50 was employed to conduct weighted mutational burden tests for each gene, with a minor allelic count (MAC) threshold set to 5 (weight ≠ 0) (See Supplementary Methods for details).

### 2.6 Pathway-based rare variant enrichment analysis

We obtained aggregated mutation burdens of gene sets related to biological processes, molecular pathways, or disease phenotypes from the following databases: BioCarta (http://www.biocarta.com/), Gene OntoManhattan plot depicting pathway-based rare variant logy (GO, http://geneontology.org/) for biological processes, Human Phenotype Ontology (HPO, https://hpo.jax.org/), Kyoto Encyclopedia of Genes and Genomes (KEGG, https://www.genome.jp/kegg/), Mouse Phenotype Ontology (MPO, https://www.mousephenotype.org/), Pathway Interaction Database (PID, http://pid.nci.nih.gov/), Reactome (https://reactome.org/), and WikiPathways (WP, https://www.wikipathways.org/). For a specific set of genes, the mutation value (equivalent to the gene-based test) for each gene was combined (for each person) into a total score. This score was then employed in a logistic regression model to estimate the likelihood of a case or control status. Enrichment in the analyzed gene sets is indicated by odds ratios and *p*-values.

### 2.7 CNV association analysis

For each detected CNV, the genomic position was matched against known gene boundaries to classify CNVs that are entirely within a gene (intragenic), span multiple genes, or lie between genes (intergenic). For each gene, CNVs from individual samples were aggregated to produce a gene-level CNV score. This was done to evaluate the collective impact of CNVs on individual genes and to facilitate gene-based association testing. Gene-based CNV scores were statistically assessed for their association with the coexistence of CS and CAKUT using the chi-square test.

## 3 Results

### 3.1 Clinical landscape of CS and CAKUT coexistence

In our study cohort, we examined a total of 40 patients with CS-CAKUT. The gender distribution in this cohort roughly follows a male-to-female ratio of 2:1. Notably, among the patients categorized under the scoliosis subtype, CS Type I exhibited the highest prevalence, constituting 40% of the entire patient population. These spinal curvatures primarily manifested within the thoracic region, specifically between vertebrae T1–T12. As for the CAKUT phenotype, unilateral renal agenesis displayed the most frequent characteristic in the cohort, affecting 32.5% of the patient cohort. These findings are summarized in Table 1.

**TABLE 1:** Distribution of clinical characteristics in all patients.

Characteristic	Patient no. (%)	
Gender	Female	32.5%
	Male	67.5%
Scoliosis subtype	CS Type I	40.0%
	CS Type II	15.0%
	CS Type III	35.0%
	IS	10.0%
Cobb angle (degree)	< 40	20.0%
	40∼80	62.5%
	≥ 80	17.5%
Spinal curve location	Cervical	10.0%
	Thoracic (T1-T12)	67.5%
	lumbar (L1-L5)	52.5%
	Sacral	5.0%
CAKUT Phenotype	Unilateral Renal agenesis	32.5%
	Horseshoe kidney	22.5%
	Duplication of renal pelvis	2.5%
	Hydronephrosis	22.5%
	Hypospadias	7.5%
	Multicystic kidney dysplasia	5.0%

Patients with multiple spinal curves are repeat counted. CS, congenital scoliosis; IS, idiopathic scoliosis; CAKUT, Congenital Anomalies of the Kidney and Urinary Tract.

**FIGURE 1:**
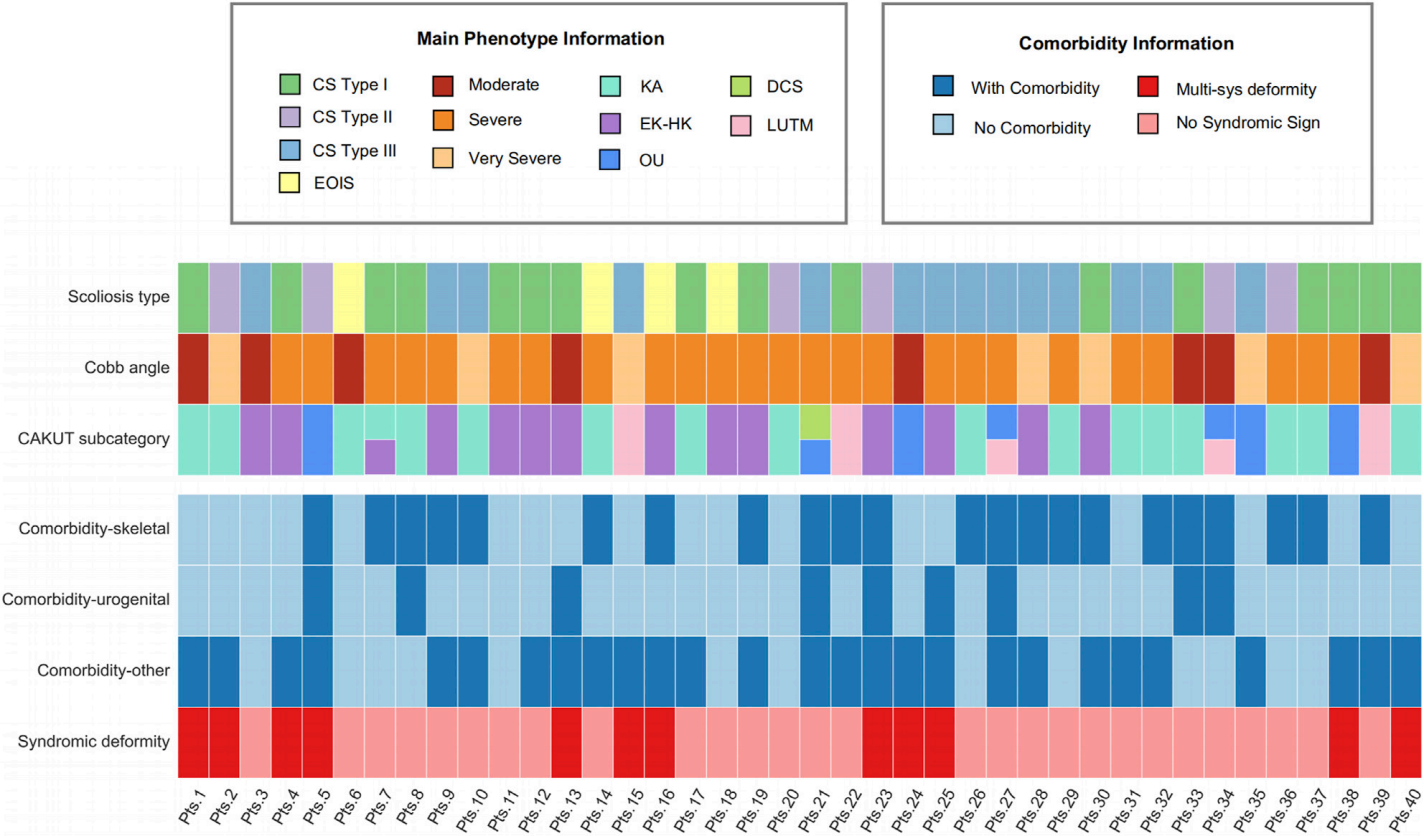
Comprehensive clinical profile heatmap of MBC patients. The heatmap provides a visualization of distinct clinical attributes associated with individual MBC patients (each represented as a separate column). It encompasses detailed phenotype data relating to scoliosis and CAKUT, complemented with associated comorbidity records. Diverse subtypes are demarcated using varying color codes for ease of identification. Pts, patients; KA, kidney anomaly; EK-HK, ectopic kidney and horseshoe kidney; OU, obstructive uropathy; DCS, duplicated collecting system and/or ureter; LUTM, other lower urinary tract malformation.

Additionally, the characterization of patient traits highlighted that those with CS Type I in conjunction with renal abnormal (KA and EK-HK) displayed a syndromic deformity frequency of 33.3%, accounting for 4 out of the 12 patients in this subgroup (Figure 1). Consequently, it is common to observe variations in other systemic phenotypes in these individuals. Moreover, among the subset of 5 patients diagnosed with OU CAKUT, 3 patients experienced syndromic deformities, constituting 60% of this subgroup. Particularly noteworthy is that 2 of these patients exhibited respiratory system phenotypes, specifically pulmonary hypoplasia or restrictive ventilatory defects. It is crucial to emphasize that both of these patients were diagnosed with CS Type III in conjunction with OU. Due to the more severe symptoms associated with CS Type III, our findings indicate that it often presents with comorbidities such as Rib Fusion and Hypoplasia of the ribs. Another notable discovery was the occurrence of dextrocardia in two KA patients. Specifically, one had CS Type I combined with a horseshoe kidney, and the other had CS Type III combined with unilateral renal agenesis. Both individuals also present with severe scoliosis and comorbidities beyond skeletal and urogenital issues (Figure 1).

In summary, our study has revealed the intricate and diverse phenotypic variations among patients with CS-CAKUT, encompassing aspects of the spine, urinary system, and other bodily systems.

### 3.2 Gene-based rare variant analysis identifies *PTPN11* as a novel candidate gene for CS-CAKUT

Building on these clinical observations, we employed a weighted gene-based burden test to compare the genome-wide frequencies of rare coding variants between CS—CAKUT cases and controls.

**FIGURE 2:**
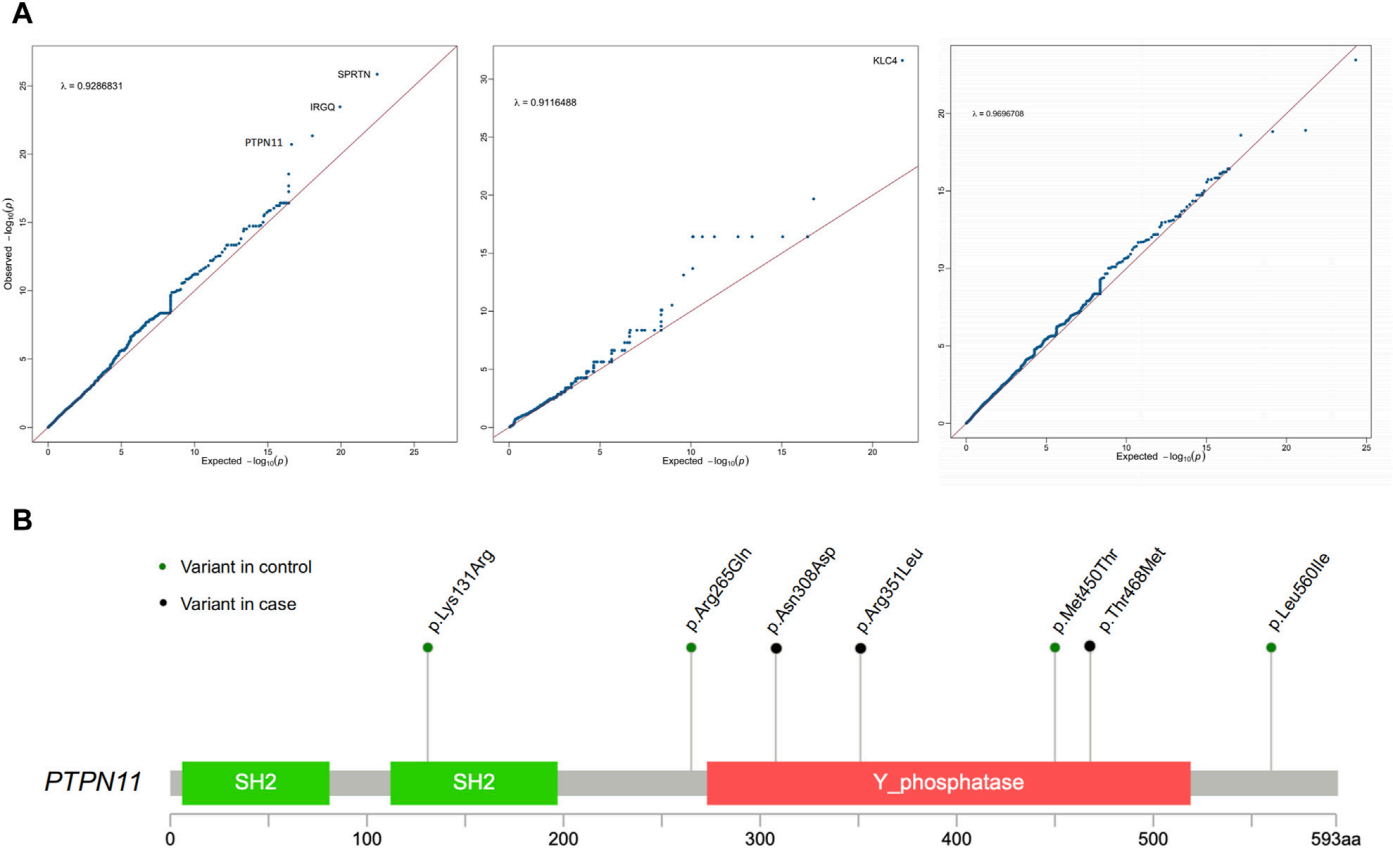
Association analysis of rare genetic variants identifies PTPN11 as a novel candidate gene for CS-CAKUT. **(A)** Quantile-quantile (Q-Q) plot of the association analysis based on rare genetic variants. The y-axis represents observed data, while the x-axis represents the expected values under a normal distribution. The plotted points indicate deviations between the observed distribution and the expected normal distribution. **(B)** Spectrum of deleterious missense (D-mis) and likely gene-disruptive (LGD) variants identified from CS-CAKUT cases in the PTPN11 gene.

In comparison to the control group, *SPRTN* exhibited the strongest correlation (*p* = 1.41E-26) (Table 2; Figure 2A). The protein encoded by *SPRTN* potentially participates in DNA repair during replication in the presence of damaged DNA. Mice lacking a similar protein display chromosomal instability and a premature aging phenotype. Mutations in this gene are associated with Ruijs-Aalfs syndrome (RJALS), characterized by intellectual developmental delay, atypical facial features, short stature, other skeletal abnormalities, and neurological issues. Previous studies have also linked *SPRTN* to premature aging and hepatocellular carcinoma (Hiom, 2014; Lessel et al., 2014). Particularly intriguing was our discovery concerning protein tyrosine phosphatase non-receptor 11 (*PTPN11*) (*p* = 1.95E-21), an intracellular protein tyrosine phosphatase (PTP) (Kamiya et al., 2014) (Table 2; Figure 2A). Germline point mutations in the *PTPN11* gene lead to Noonan syndrome (NS; OMIM 163950) and LEOPARD syndrome (LS; OMIM 151100), both sharing several common features, notably skeletal abnormalities, consistent with our study cases. Therefore, *PTPN11* emerges as a compelling candidate gene for CS-CAKUT.

**TABLE 2:** Top 10 genes identified by gene-based rare variant analysis.

Rank	Gene	Case.masks	Control.masks	Burden norminal OR (95% CI)	*p* value	*p* value
1	SPRTN	2/39:1_mask2|1_mask4m	2/2764:1_mask5|1_mask5m	1.41E-26	0.00537722	165 (9.74–2780)
2	IRGQ	2/39:1_mask1|1_mask4m	6/2764:6_mask5m	3.50E-24	0.009353846	118 (10.8–1300)
3	MOB2	2/39:1_mask4m|1_mask5m	2/2764:2_mask5m	4.57E-22	0.010363375	90.9 (3.94–2100)
4	PTPN11	3/39:2_mask2m|1_mask5m	5/2764:1_mask2m|1_mask3m|3_mask5m	1.95E-21	0.010404332	76.6 (11.2–521)
5	C2orf73	2/39:1_mask1|1_mask2	13/2764:1_mask1|1_mask5|11_mask5m	2.88E-19	0.010562225	62.4 (10.4–373)
6	MRPS35	3/39:1_mask2|1_mask3m|1_mask4m	9/2764:1_mask2|1_mask3m|4_mask4m|3_mask5m	2.10E-18	0.010663846	46.4 (7.98–269)
7	CLDN3	2/39:1_mask2m|1_mask4m	6/2764:4_mask4m|2_mask5m	5.60E-18	0.011965967	60.6 (7.22–508)
8	C9orf163	1/39:1_mask2	0/2764	3.76E-17	0.012327097	186 (7.01–4920)
9	CLDN24	1/39:1_mask2	0/2764	3.76E-17	0.018556476	186 (7.01–4920)
10	HIST3H2A	1/39:1_mask5m	0/2764	3.76E-17	0.021565566	84.2 (1.95–3640)

The table shows the top 10 genes identified in the analysis, along with the minor allele counts and variant mask levels for each gene. Odds ratios were calculated for cases versus in-house controls.

The burden *p*-values were calculated using the weighted burden test and the norminal *p*-values were calculated using the Fisher's exact test.

All identified deleterious variants, p. Asn308Asp, p. Arg351Leu, and p. Thr468Met, in CS-CAKUT patients are located within the Phosphatase domain (Figure 2B). This domain plays a vital role in promoting extracellular matrix mineralization and is linked to hypophosphatasia (Yadav et al., 2011). The three patients carrying loss-of-function variants exhibit severe scoliosis and KA, with additional phenotypes mainly affecting the cardiovascular and pulmonary domains. Notably, one of these patients has dextrocardia. Previous reports have closely associated *PTPN11* p. Asn308Asp with Noonan syndrome, characterized by typical facial features, cardiac issues, growth problems (Beneteau et al., 2009), and varying cognitive impairments, aligning with our observed phenotypes. Furthermore, *PTPN11* p. Thr468Met has been found to cause not only Noonan syndrome but also exhibit a close connection with LEOPARD syndrome (Conboy et al., 2016; Zhang et al., 2016). This highlights the significant role of *PTPN11* in skeletal growth and remodeling.

### 3.3 Cell signaling transduction and mitosis are critical pathways in CS-CAKUT

**FIGURE 3:**
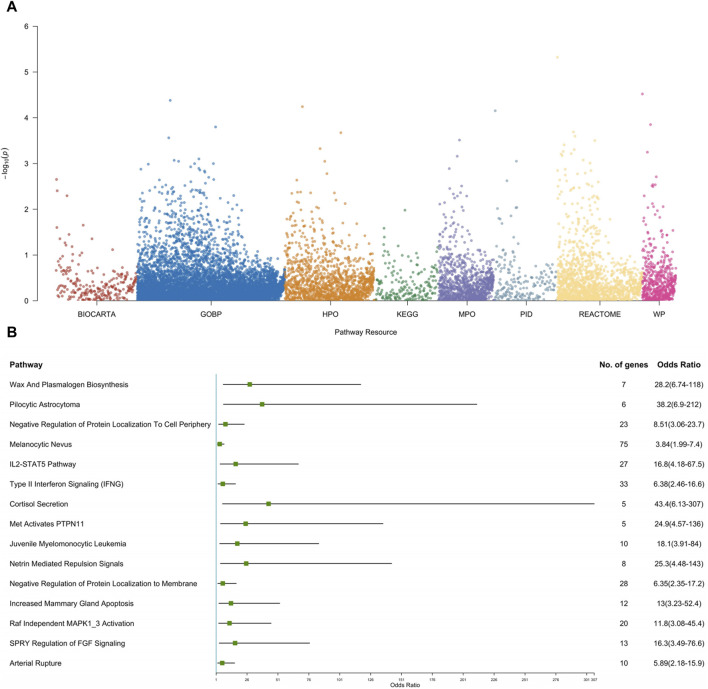
Pathway-based gene set burden analysis elucidates the pathogenic mechanisms involved in CS-CAKUT. **(A)** Manhattan plot depicting pathway-based rare variant associations. The y-axis represents the -log10 p-values for pathways, while the x-axis indicates the resources used for analysis, including BioCarta, Gene Ontology Biological Process (GOBP), Human Phenotype Ontology (HPO), Kyoto Encyclopedia of Genes and Genomes (KEGG), Mouse Phenotype Ontology (MPO), Pathway Interaction Database (PID), Reactome, and WikiPathways (WP). **(B)** Forest plot of the top 15 pathways based on rare variant association results. Each square represents the odds ratio (OR) for each pathway, and the horizontal line represents the corresponding 95% confidence interval (CI).

In our effort to thoroughly investigate functional pathways enriched with genetic variations, we conducted a comprehensive analysis using the pathway-based gene burden approach. Figure 3 meticulously delineates the top 15 pathways that emerged as significant contributors in our study.

Among these pathways, three stood out due to their significant enrichment in cell signaling mechanisms: “Netrin Mediated Repulsion Signals (OR = 25.3, *p* = 0.0003),” “SPRY Regulation of FGF Signaling (OR = 16.3, *p* = 0.0004),” and “Raf Independent MAPK1_3 Activation (OR = 11.8, *p* = 0.0003) (Supplementary Table S2).” These findings hold substantial importance as they are closely tied to cell signaling cascades crucial for the activation of the Mitogen-Activated Protein Kinase (MAPK) pathway. The centrality of the MAPK/ERK pathway cannot be overstated, as it plays a pivotal role in orchestrating the intricate process of ureteric bud (UB) branching morphogenesis—a fundamental aspect determining the kidney’s size, structure, and the composition of its renal units (Kurtzeborn et al., 2019). Additionally, the tyrosine phosphatase SHP2, encoded by the *PTPN11* gene, is essential for MAPK cascade activation (Roberts et al., 2007). A prior study by Du and colleagues demonstrated that Robo2 and Gen1 jointly regulate UB initiation, significantly increasing the incidence of CAKUT. Importantly, the MAPK/ERK pathway serves as a central mechanism for these two genes (Du et al., 2023). In line with Du’s findings, our study suggests that *PTPN11* may potentially influence UB development leading to CAKUT through the aforementioned three putative cell signaling pathways, in addition to modulating the MAPK/ERK pathway.

This discovery illuminates the intricate interplay between these pathways and the development of both the spine and the kidneys, providing valuable insights into the complex molecular mechanisms governing the morphogenesis of these vital structures. For a more intuitive understanding of these pivotal pathways, please refer to Figures 3A, B, which offer compelling insights into the potential keys for unraveling the complexities of spine and kidney development and function.

### 3.4 Rare CNVs association expanded the shared genetic architecture

To obtain a wide genomic understanding of the genetic pathogenisis of CS-CAKUT, we performed association analysis of CNVs that are most likely to disrupt gene function.

Our findings revealed significant copy number loss signals in *NTRK3* (*p* = 1.29E-05) and *KMT2B* (*p* = 3.26E-06) (Supplementary Table S3). *NTRK3* encodes the neurotrophic tyrosine receptor kinase (NTRK), and *KMT2B* is responsible for encoding histone H3 methyltransferase. Both genes have the potential to influence intracellular phosphorylation signaling pathways, regulating crucial protein phosphorylation and dephosphorylation events. These pathways are vital across various biological processes and have implications for conditions such as childhood muscle tone disorders and congenital mesoblastic nephroma (CMN) (Knezevich et al., 1998; Meyer et al., 2017).

Additionally, copy number loss of *IGFLR1* (*p* = 3.26E-06) emerges as a noteworthy result (Supplementary Table S3). *IGFLR1*, also known as Transmembrane Protein 149 (*TMEM149*), is a protein-encoding gene located on chromosome 19. It is widely expressed in lymph nodes, spleen, and kidneys and has been implicated in promoting the progression of renal cell carcinoma (Fagerberg et al., 2014; Song et al., 2020). Despite its relatively limited attention in previous research, this uncharacterized gene possesses structural characteristics akin to the tumor necrosis factor receptor family. Tumor necrosis factor, in turn, plays a significant role in spinal deformities (Kudo et al., 2018). Our analysis has suggested that haploinsufficiency of *IGFLR1* might hold a close association with the pathogenesis of CS-CAKUT, shedding new light on a previously underexplored genetic component within this complex syndrome.

## 4 Discussion

In the intricate landscape of congenital anomalies, the concurrent presentation of CS and CAKUT represents an enigmatic juxtaposition (Verbitsky et al., 2019; Yang et al., 2020), necessitating a deep dive into their potentially shared genetic foundation. Our study, embracing this challenge, yields pivotal insights into the genes and pathways that might intertwine these conditions.

The marked male predominance in our cohort suggests possible sex-linked genetic factors or embryonic hormonal modulations. The significance of this trend, however, warrants further investigation. The prevalence and association of CS Type I and KA, which are both simply dysplasia or agenesis, points to the dysfunction in the early developmental stages that determine the organogenesis. Moreover, a notable anomaly in our data is the elevated occurrence of dextrocardia (2/40), starkly contrasting the reported incidence of 1 in 9,00,000 in adults (Leung and Robson, 2006; Bohun et al., 2007). These dextrocardia cases coincide with CS Type III and EK-HK diagnoses, which are involved with an impaired spatial development, implicating a disrupted left-right determination could potentially underpin the pathogenesis of these conditions, indicating especially severe phenotypes.

Unraveling the genetic layers, while the association of *SPRTN* aligns with its recognized functions, the centrality of *PTPN11* emerges as a revelation. Given its links to Noonan and LEOPARD syndromes, conditions punctuated by distinct skeletal perturbations, the spotlight on *PTPN11* seems justified. Intriguingly, specific mutations within its Phosphatase domain might be the molecular culprits behind the phenotypic manifestations observed in our patients. *PTPN11*, with its product SHP2 playing a cardinal role in the MAPK cascade, emerges as the potential nexus. Our investigation into the pathway enrichment further illuminated the indispensable role of the MAPK/ERK pathway, supporting the initial findings. Moreover, CNV analysis added depth to our genetic understanding, highlighting genes such as *NTRK3*, *KMT2B*, and *IGFLR1*. Associations of these genes suggest a wider genetic dysregulation, potentially impacting signaling pathways and even epigenetic modifiers.

Our study, while illuminating, has limitations. Primarily, our focus on the Han Chinese population may limit the generalizability to other ethnic groups. The modest sample size could hinder the detection of rare variants with subtle effects. While we highlighted genes and pathways of interest, we did not definitively established causality. Furthermore, interactions between genes and potential environmental factors remain largely unexplored in our analysis. Future studies with diverse cohorts are essential to build on our findings.

## 5 Conclusion

Taken together, our findings paint a picture of a multifaceted genetic landscape underpinning the CS-CAKUT coexistence. The clinical and genomic data converge towards shared developmental pathways, with genes such as *PTPN11* playing pivotal roles. While our study offers a significant insight in comprehensive understanding of these conditions, it also underscores the need for further investigations, especially targeting the identified pathways and genes, to elucidate the full mechanistic insights and potentially pave the way for therapeutic interventions.

## Data Availability

The data that support the findings of this study are available from the corresponding author upon reasonable request, subject to permission from the ethics committee and the provisions of the relevant data protection regulations. The data will be made publicly available in a repository at the conclusion of the ongoing research project.
