# Supplementary material for: Deciphering the genomic insights into the coexistence of congenital scoliosis and congenital anomalies of the kidney and urinary tract
Source: Front Genet. 2024 Jul 23;15:1399604. doi: 10.3389/fgene.2024.1399604 (PMC11300289; doi:10.3389/fgene.2024.1399604)
Supplement: Supplementary file 1 [file Table1.DOCX]

**Supplementary Methods**

### Basic quality control and processing of sequencing data

The raw sequencing data were initially subjected to basic quality control (QC) through FastQC and trimming. The resulting high-quality sequencing data were then aligned to the GRCh38 human reference genome using Picard (broadinstitute.github.io/picard/) and BWA[^4^](#_ENREF_4) to generate Binary Alignment Map (BAM) files. The conversion of BAM files to the genomic variant call file (gVCF) was executed using the DNAseq module of the Sentieon software[^5^](#_ENREF_5). Subsequently, a joint call for SNVs and small insertions/duplications (indels) was performed by combining the individual gVCFs using the Sentieon tool.

### Advanced variant-level QC

SNVs and indels underwent multiple layers of filtration:

1. Hard filter: We filtered out variants that meet any of the following criteria:
   1. genotype quality (GQ) < 20
   2. depth (DP) < 10
   3. quality by depth (QD) < 2
   4. strand odds ratio (SOR) > 9
   5. Variant allele balance < 25%
2. Population-based filter: We filtered out variants that deviated from the Hardy-Weinberg equilibrium (p < 0.000001) and variants with a missing rate > 10% in the case-control population.
3. Variant Quality Score Recalibration (VQSR) was performed using the standard GATK protocol with a sensitivity of 99%.

CNVs were filtered out according to the following criteria:

1. Abnormal filter flag generated by CNVpytor indicating reductant reads mapped in the CNV region (q0 > 0.5).
2. P-value calculated using t-test statistics between RD statistics in the region and global was not significant (e-val1> 0.05).
3. Reciprocal overlap proportion with RLCRs > 70%

To note, the RLCR definition was including assembly gaps and segmental duplications from UCSC Genome Browser database (<http://genome.ucsc.edu>, accessed on 2022/10/3). The RepeatMasker regions were excluded from the definition of RLCR due to a prior study suggesting that filtering based on RepeatMasker did not reduce false discovery rate (FDR), but only slightly reduced sensitivity (by approximately 5%) for rare, genic CNVs[^6^](#_ENREF_6).

### Sample -level QC

After variant-level QC, we performed ancestry estimation by principal component analysis (PCA) using plink (version 1.90) based on the combined genotypes of the in-house subjects and the 1000 Genomes Phase III population^1^. Samples that met any of the following criteria were excluded:

1. PCA outliers ( > 2 standard deviation)
2. Overall call rate < 0.95
3. Average depth < 30X
4. Heterozygosity < 0.8

In addition, the relatedness among individuals was calculated using the identity-by-descent (IBD) analysis. For each pair of individuals with IBD > 0.8, we excluded the one with a lower call rate.

## Gene-based association analysis

**Weighting of variants**

A weight of 0-1 was assigned to each variant according to the variant type and bioinformatic predictions. REVEL^2^ and CADD, two ensemble predictors, were used for the prediction of missense variants and in-frame indels respectively. The detailed weighting standards are provided in Supplementary Table 1.

**Subset analysis**

In addition to a global weighted burden test, we also performed on a subset of synonymous variants to calibrate the burden test. For the synonymous variants, a minimum variant count of n=3 in the case-control population was required for a gene to be included rather than n=5 for the all-variant-model.

**Statistic methods**

After variant filtration, the number of cases/controls carrying at least one qualifying variant in each gene was calculated and compared using a two-sided Fisher’s Exact test. P-values were adjusted for multiple testing using the Benjamini-Hochberg (BH) procedure. A conservative Bonferroni-corrected gene-level exome-wide significance threshold of P = 0.05/ (1 model × 19,337 genes) = 2.6× 10^-6^ was used.

**Supplementary Table 1 The detailed weighting standards**

| **Mask Level** | **­­­Weight Value** | **LOF variants*** | **Non-canonical splicing variants** | **In-frame indels** | **Missense variants** |
| --- | --- | --- | --- | --- | --- |
| **Mask 1** | 1 | Labeled as ‘high confidence’ by LOFTee | - | - | - |
| **Mask 2** | 0.8 | Labeled as ‘low confidence’ or unlabeled by LOFTee | SpliceAI > 0.5 | - | REVEL > 0.8 |
| **Mask 3** | 0.6 | - | - | CADD score > 20 | REVEL > 0.6 |
| **Mask4** | 0.4 | - | - | CADD score > 10 | REVEL > 0.4 |
| **Mask5** | 0.2 | - | - | Others | REVEL > 0.2 |
| **Mask6** | 0 | - | - | - | Others |
| The detailed weighting standards | | | | | |

**Supplementary Table 2** Top 15 signal of pathway

| Pathway | Source | No. of genes | Case.num | Odds Ratio | P-value |
| --- | --- | --- | --- | --- | --- |
| Wax And Plasmalogen Biosynthesis | REACTOME | 7 | 4/39 | 28.2(6.74-118) | 4.77e-06 |
| Pilocytic Astrocytoma | WP | 6 | 4/39 | 38.2(6.9-212) | 3.01e-05 |
| Negative Regulation of Protein Localization To Cell Periphery | GOBP | 23 | 8/39 | 8.51(3.06-23.7) | 4.16e-05 |
| Melanocytic Nevus | HPO | 75 | 24/39 | 3.84(1.99-7.4) | 5.72e-05 |
| IL2-STAT5 Pathway | PID | 27 | 8/39 | 16.8(4.18-67.5) | 7.03e-05 |
| Type II Interferon Signaling (IFNG) | WP | 33 | 11/39 | 6.38(2.46-16.6) | 1.41e-04 |
| Cortisol Secretion | GOBP | 5 | 3/39 | 43.4(6.13-307) | 1.58e-04 |
| Met Activates PTPN11 | REACTOME | 5 | 4/39 | 24.9(4.57-136) | 2.04e-04 |
| Juvenile Myelomonocytic Leukemia | HPO | 10 | 5/39 | 18.1(3.91-84) | 2.12e-04 |
| Netrin Mediated Repulsion Signals | REACTOME | 8 | 9/39 | 25.3(4.48-143) | 2.52e-04 |
| Negative Regulation of Protein Localization to Membrane | GOBP | 28 | 8/39 | 6.35(2.35-17.2) | 2.74e-04 |
| Increased Mammary Gland Apoptosis | MPO | 12 | 8/39 | 13(3.23-52.4) | 3.07e-04 |
| Raf Independent MAPK1_3 Activation | REACTOME | 20 | 6/39 | 11.8(3.08-45.4) | 3.14e-04 |
| SPRY Regulation of FGF Signaling | REACTOME | 13 | 5/39 | 16.3(3.49-76.6) | 3.90e-04 |
| Arterial Rupture | REACTOME | 10 | 12/39 | 5.89(2.18-15.9) | 4.73e-04 |

Top 15 signal of pathway based association result was shown.

**Supplementary Table 3** Top 10 signal of CNV

| Gene | CNV type | Case count | Control  count | Odds ratio | P value |
| --- | --- | --- | --- | --- | --- |
| *RBMS2* | Deletion | 3/40 | 0/2764 | inf | 3.26e-06 |
| *IGFLR1* | Deletion | 3/40 | 0/2764 | inf | 3.26e-06 |
| *KMT2B* | Deletion | 3/40 | 0/2764 | inf | 3.26e-06 |
| *NTRK3* | Deletion | 3/40 | 1/2764 | 222.35 | 1.29e-05 |
| *RFX2* | Deletion | 4/40 | 12/2764 | 26.02 | 6.99e-05 |
| *TCF3* | Deletion | 3/40 | 4/2764 | 55.45 | 0.00010967 |
| *RNF38* | Deletion | 3/40 | 4/2764 | 55.45 | 0.00010967 |
| *BLOC1S2* | Deletion | 2/40 | 0/2764 | inf | 0.00023175 |
| *OLMALINC* | Deletion | 2/40 | 0/2764 | inf | 0.00023175 |

Top 10 signal of CNV association result was shown. Inf, infinite.
